# Supplementary material for: Antidepressive-like Behavior-Related Metabolomic Signatures of Sigma-1 Receptor Knockout Mice
Source: Biomedicines. 2022 Jul 1;10(7):1572. doi: 10.3390/biomedicines10071572 (PMC9313356; doi:10.3390/biomedicines10071572)
Supplement: Supplementary file 1 [file biomedicines-10-01572-s001.zip › biomedicines-1754556-supplementary.pdf]

Supplementary Table S1. Measured and detected metabolites using MxP® Quant 500 kit for targeted metabolic profiling in mouse plasma and brain tissue.

Biochemical classes	Measured metabolites	Detected metabolites PLASMA	BRAIN
Alkaloids	**1**	1	0
Amine Oxides	**1**	1	0
Amino Acids	**20**	20	20
Amino Acid Related	**30**	23	12
Bile Acids	**14**	6	0
Biogenic Amines	**9**	6	6
Carbohydrates and Related	**1**	1	0
Carboxylic Acids	**7**	3	4
Cresols	**1**	1	0
Fatty Acids	**12**	3	4
Hormones and Related	**4**	0	0
Indoles and Derivatives	**4**	3	0
Nucleobases and Related	**2**	0	2
Vitamins and Cofactors	**1**	1	1
Acylcarnitines	**40**	8	14
Lysophosphatidylcholines	**14**	8	7
Phosphatidylcholines	**76**	72	66
Sphingomyelins	**15**	13	11
Ceramides	**28**	7	13
Dihydroceramides	**8**	0	0
Hexosylceramides	**19**	4	13
Dihexosylceramides	**9**	2	1
Trihexosylceramides	**6**	0	0
Cholesteryl Esters	**22**	10	0
Diglycerides	**44**	10	5
Triglycerides	**242**	170	0
**TOTAL**	**630**	**373**	**179**

Supplementary Table S2. Primer sequences of the genes used in this study.

Gene symbol	Full name	NCBI Accession number	Forward primer sequence (5'->3')	Reverse primer sequence (5'->3')	Amplicon length, b
Actb	Actin, beta	NM_007393.5	CCTCTATGCCAACACAGTGC	CATCGTACTCCTGCTTGCTG	215
Dnajc14	DnaJ heat shock protein family (Hsp40) member C14	NM_001359825.1	AGTGACCCGACTCTTGACCA	TCTGCCATCCGTTTCATCTCA	249
Drd1	Dopamine receptor D1	NM_010076.3	GTCTCCCAGATCGGGCATTT	CAGTCACTTTTCGGGGATGC	111
Drd2	dopamine receptor D2	NM_010077.3	ATCGTCTCGTTCTACGTGCC	TGGGTACAGTTGCCCTTGAG	155
Gabbr2	gamma-aminobutyric acid (GABA) B receptor, 2	NM_001081141.2	ATCGAGCAGATCCGCAACGAG	ACACAACTTGACCCGTGAC	997
Htr1a	5-hydroxytryptamine (serotonin) receptor 1A	NM_008308.4	CCCCCTTTCAAGGAACAGCA	GAGCCCAGAGATCTGCGAAA	102
Htr1b	5-hydroxytryptamine (serotonin) receptor 1B	NM_010482.2	GGAGCGCAAAGACTCAAAGC	TCTCAGGTTCCCTTGTCCCT	116
Htr2a	5-hydroxytryptamine (serotonin) receptor 2A	NM_172812.3	TCCTGTATGGGTACCGGTGG	ATGGTCCACACCGCAATGAT	202
Htr2c	5-hydroxytryptamine (serotonin) receptor 2C	NM_008312.4	GGACGCTAGTGGGTTGTCAC	GGCCAATTAGGTGCACAAGG	189

Htr3a	5-hydroxytryptamine (serotonin) receptor 3A	NM_013561.2	GATGACTGCTCAGGTTCTGATCTT	GTGGTGGAAGAGGGCTACCT	115
Pgrmc1	Progesterone receptor membrane component 1	NM_016783.4	GGCAAGGTGTTCGACGTGA	GTCCAGGCAAAATGTGGCAA	111
Sigmar1	Sigma non-opioid intracellular receptor 1	NM_011014.3	CATTCGGGACGATACTGGGC	CCTGGGTAGAAGACCTCACTTTT	101
Sigmar1	Sigma non-opioid intracellular receptor 1	NM_011014.3	CTCGCTGTCTGAGTACGTG	AAGAAAGTGTCGGCTAGTGCAA	186/279
Tph2	Tryptophan hydroxylase 2	NM_173391.3	CTCACCGAGTCCTCATGTACG	TGGCCACATCCACAAAATACTTC	102

Supplementary Table S3. Significantly altered metabolite in adult Sig1R KO mice plasma.

ADULT mice PLASMA				
**Group**	**Metabolite**	**Concentration (µM)**	**Down or Up**	***P* value**
Amino acids	Alanine (Ala)	286.9	Up	0.006
	Arginine (Arg)	72.9	Up	0.003
	Asparagine (Asn)	26.2	Up	0.002
	Glutamine (Gln)	460.5	Up	0.022
	Glutamate (Glu)	20.1	Down	0.041
	Glycine (Gly)	214	Down	0.014
	Histidine (His)	76.7	Down	0.003
	Isoleucine (Ile)	47.6	Down	0.001
	Leucine (Leu)	72.1	Down	0.001
	Lysine (Lys)	235	Down	0.015
	Methionine (Met)	38.4	Down	4.84E-05
	Phenylalanine (Phe)	56.6	Down	0.001
	Proline (Pro)	72.7	Down	0.004
	Serine (Ser)	102.7	Up	0.046
	Threonine (Thr)	107.3	Down	5.71E-05
	Valine (Val)	135.3	Down	4.56E-05
Amino acids related	alpha-AAA	1.3	Down	0.004
	Asymmetric dimethylarginine (ADMA)	0.7	Up	0.044
	Anserine	0.6	Up	0.002
	beta-aminobutyric acid (BABA)	0.3	Down	0.046
	Carnosine	0.4	Up	0.045
	Citrulline	63.3	Up	0.001
	Methionine sulfoxide	2.2	Down	0.003
	Ornithine	59.1	Up	0.018
	Sarcosine	1.1	Down	1.63E-04
	Taurine	196.7	Down	0.025
Bile Acids	Taurocholic acid (TCA)	0.027	Down	0.011
	Tauromurocholic acid (TMCA)	0.026	Down	0.045
Biogenic Amines	GABA	0.128	Up	0.034
	Histamine	2	Up	0.004
Carboxylic Acids	Hippuric acid	2.4	Down	0.001
	Lactic acid	3936.2	Down	0.042
	Succinic acid	18	Up	0.039
Indoles and Derivatives	3-Indoleacetic acid	0.3	Up	0.036
	3-Indolepropionic acid	2.3	Up	0.016

	Indoxyl sulfate	4.6	Down	0.009
Acylcarnitines	Acetylcarnitine (C2)	5.2	Down	2.19E-05
	Propionylcarnitine (C3)	0.17	Down	1.47E-04
	Butyrylcarnitine (C4)	0.24	Up	5.08E-04
	Valerylcarnitine (C5)	0.08	Down	0.016
Lysophosphatidylcholines	lysoPC a C16:0	134.8	Down	1.64E-04
Phosphatidylcholines	PC aa C30:2	0.39	Up	0.041
	PC aa C32:0	9.55	Down	3.11E-05
	PC aa C32:1	12.63	Down	0.003
	PC aa C32:3	0.26	Up	0.023
	PC aa C34:1	177.3	Down	0.002
	PC aa C34:2	314.7	Down	0.006
	PC aa C34:3	11.8	Up	0.050
	PC aa C36:1	30.5	Down	0.012
	PC aa C36:3	116.4	Down	0.029
	PC aa C36:4	161.3	Up	0.050
	PC aa C36:5	6	Up	0.040
	PC aa C38:1	1.1	Down	0.028
	PC aa C42:0	0.16	Down	0.009
	PC ae C34:1	5	Down	0.014
	PC ae C36:0	0.4	Up	0.022
	PC ae C36:1	3.7	Up	0.023
	PC ae C38:1	1.4	Down	0.012
	PC ae C38:2	9.7	Down	0.014
	PC ae C40:2	0.42	Down	0.034
	PC ae C40:6	1.84	Up	0.046
	PC ae C42:1	0.37	Up	0.045
Sphingomyelins	SM (OH) C14:1	1.23	Up	0.009
	SM C16:0	13.3	Down	0.007
	SM C16:1	3	Up	0.039
	SM C24:0	5.8	Down	0.007
Ceramides	Cer(d18:1/16:0)	0.06	Down	0.006
	Cer(d18:1/23:0)	0.191	Down	0.004
	Cer(d18:1/24:0)	0.417	Down	0.001
	Cer(d18:1/24:1)	0.437	Up	0.023
Cholesteryl esters	CE(16:0)	56.4	Down	0.007
	CE(18:2)	439.9	Down	0.035
Diglycerides	DG(18:1_18:2)	9.1	Up	0.045
Triglycerides	TG(16:0_32:0)	18.4	Down	0.019
	TG(16:0_32:1)	35.5	Down	0.023
	TG(16:0_34:0)	13.2	Down	0.040
	TG(16:0_34:1)	128.9	Down	0.041
	TG(16:1_32:0)	12	Down	0.019

	TG(18:0_32:1)	2.5	Down	0.041
	TG(20:3_36:3)	3.9	Up	0.039
	TG(22:5_34:2)	7.9	Up	0.043
	TG(22:5_34:3)	0.9	Up	0.017

Supplementary Table S4. Significantly altered metabolite in old Sig1R KO mice plasma.

OLD mice PLASMA				
**Group**	**Metabolite**	**Concentration (μM)**	**Down or Up**	***P* value**
Amine oxides	TMAO	5.8	Up	0.029
Amino acids	Methionine (Met)	41.2	Down	0.038
Amino acids related	alpha-AAA	1.7	Down	0.043
	beta-aminobutyric acid (BABA)	0.4	Up	0.047
	Kynurenine	1.4	Up	0.005
	Phenylacetylglycine (PAG)	3.8	Up	0.025
Biogenic amines	Histamine	3.1	Up	0.002
	Serotonin	0.4	Up	0.028
Carboxylic acids	Hippuric acid	6.4	Up	8.17E-04
Indoles and derivates	Indoxyl sulfate	14.1	Up	0.003
Acylcarnitines	Acetylcarnitine (C2)	6.3	Up	0.025
	Butyrylcarnitine (C4)	0.31	Up	0.028
	Valerylcarnitine (C5)	0.08	Up	0.045
Lysophosphatidylcholines	lysoPC a C16:0	119.8	Up	0.004
	lysoPC a C16:1	4.8	Down	0.030
	lysoPC a C18:1	49.8	Down	0.005
	lysoPC a C20:3	8	Down	0.038
Phosphatidylcholines	PC aa C32:0	9.38	Down	0.030
	PC aa C32:1	7.61	Down	0.027
	PC aa C34:1	129.1	Down	0.008
	PC aa C34:4	0.5	Down	0.038
	PC aa C36:1	21.6	Down	0.011
	PC aa C36:4	118.8	Down	0.040
	PC aa C38:3	26.2	Down	0.049
	PC aa C38:4	72.2	Up	0.046
	PC aa C38:5	54.7	Up	0.014
	PC aa C42:4	0.13	Up	0.011
	PC ae C36:0	0.3	Down	0.009
	PC ae C38:5	2.7	Down	0.007
	PC ae C38:6	0.98	Down	0.040
	PC ae C44:6	0.12	Down	0.003
Sphingomyelins	SM C16:0	11.3	Down	0.025
	SM C16:1	1.6	Down	0.014
Ceramides	Cer(d18:1/16:0)	0.06	Down	0.004
	Cer(d18:1/24:1)	0.492	Down	1.57E-04
Hexosylceramides	HexCer(d18:1/22:0)	1.2	Up	0.045

	HexCer(d18:1/24:0)	0.2	Down	6.48E-04
	HexCer(d18:1/24:1)	1.5	Down	8.91E-07
Dihexosylceramides	Hex2Cer(d18:1/24:1)	0.157	Down	0.004
Cholesteryl esters	CE(14:0)	3.2	Down	0.036
	CE(20:4)	430.9	Up	0.012
	CE(22:6)	52.1	Up	0.023
Diglycerides	DG(17:0_18:1)	1.1	Up	0.013
Triglycerides	TG(20:4_36:4)	1.5	Up	0.039

Supplementary Table S5. Significantly altered metabolite in adult Sig1R KO mice brain.

ADULT mice BRAIN				
**Group**	**Metabolite**	**Concentration (pmol/mg tissue)**	**Down or Up**	***P* value**
Amino acids	Ala	1001.9	Down	0.013
	Thr	363.9	Down	6.15E-04
Amino Acids related	alpha-AAA	45.6	Down	0.018
	Citrulline	11.6	Down	0.020
	Cystine	2.1	Up	0.034
	3-Met-His	3.6	Up	0.023
Fatty acids	Arachidonic acid (AA)	350.9	Down	0.033
Phosphatidylcholines	PC aa C36:1	206.2	Down	0.035
	PC aa C38:1	2.9	Up	4.31E-04
	PC aa C40:2	1.3	Up	3.89E-04
	PC aa C42:1	0.4	Up	0.001
	PC aa C42:2	0.7	Up	2.69E-05
	PC ae C36:1	9.9	Down	0.015
	PC ae C36:2	3.9	Down	0.016
	PC ae C36:3	1.6	Up	0.028
	PC ae C38:1	1.3	Up	0.012
	PC ae C38:2	1.1	Up	0.002
	PC ae C38:3	1.1	Down	0.015
	PC ae C40:2	0.5	Up	0.031
	PC ae C40:4	1.1	Up	0.020
	PC ae C40:5	1.2	Up	0.009
	PC ae C42:2	1.9	Up	0.029
	PC ae C44:6	0.4	Up	0.039
Sphingomyelins	SM C18:1	24.82	Down	0.017
	SM C24:0	3.0	Down	0.024
Ceramides	Cer (d18:1/22:0)	0.26	Up	0.012
	Cer (d18:1/24:0)	0.35	Up	0.004
	Cer (d18:1/24:1)	4.93	Up	0.004
	Cer (d18:1/25:0)	1.88	Up	0.019
Hexosylceramides	HexCer (d18:1/20:0)	1.23	Up	0.037
	HexCer (d18:1/22:0)	7.89	Up	0.008
	HexCer (d18:1/24:0)	2.47	Up	0.014
	HexCer (d18:1/24:1)	91.99	Up	0.002
Dihexosylceramides	Hex2Cer (d18:1/18:0)	2.02	Up	0.037

Supplementary Table S6. Significantly altered metabolite in old Sig1R KO mice brain.

OLD mice BRAIN				
**Group**	**Metabolite**	**Concentration (pmol/mg tissue)**	**Down or Up**	***P* value**
Amino acids	Phenylalanine (Phe)	83.7	Up	0.043
	Threonine (Thr)	314.1	Down	0.005
	Tryptophan (Trp)	23.8	Up	0.005
	Tyrosine (Tyr)	105.2	Up	0.003
Amino acids related	Anserine	6.5	Up	0.020
	SDMA	0.477	Up	0.013
Carboxylic acids	Aconitic Acid	7.7	Up	0.020
Vitamins and cofactors	Choline	287.6	Down	0.020
Acylcarnitines	Carnitine (C0)	99.1	Up	0.001
	Acetylcarnitine (C2)	37	Up	0.010
	Propionylcarnitine (C3)	1.51	Up	0.012
	Hydroxybutyrylcarnitine (C4-OH)	0.77	Up	0.007
	Butyrylcarnitine (C4)	1.365	Up	0.010
	Hydroxyvalerylcarnitine (C5-OH)	1.12	Up	0.018
	Tetradecanoylcarnitine (C14)	0.83	Down	0.010
	Hexadecanoylcarnitine (C16)	1.4	Down	0.007
	Hexadecenoylcarnitine (C16:1)	0.4	Down	0.016
	Octadecenoylcarnitine (C18:1)	1	Down	0.001
Lysophosphatidylcholines	lysoPC a C20:4	6	Down	0.004
Ceramides	Cer(d18:1/18:0)	2.65	Up	0.027
Hexosylceramides	HexCer (d18:1/18:0)	4.08	Up	0.039
	HexCer (d18:1/22:0)	7.74	Up	0.047
	HexCer (d18:2/24:0)	2.75	Up	0.016
Dihexosyl-ceramides	Hex2Cer (d18:1/18:0)	2.39	Up	0.011
Diglycerides	DG (17:0_18:1)	4.21	Up	0.037

Supplementary Table S7. Result from Pathway Analysis.

Pathway name	Total	Hits	Raw p	Holm p	FDR p	Impact
Phenylalanine metabolism	12	4	1.91E-6	8.20E-5	8.19E-5	0.36
Valine, leucine and isoleucine biosynthesis	8	4	2.19E-5	9.19E-4	4.70E-4	0.0
Aminoacyl-tRNA biosynthesis	48	20	1.59E-4	6.53E-3	2.29E-3	0.17
Valine, leucine and isoleucine degradation	40	3	3.16E-4	0.013	3.40E-3	0.0
Histidine metabolism	16	7	5.36E-4	0.021	4.30E-3	0.55
Glycerophospholipid metabolism	36	3	6.18E-4	0.023	4.30E-3	0.14
Glycine, serine and threonine metabolism	34	7	7.00E-4	0.026	4.30E-3	0.64
Arginine biosynthesis	14	6	1.85E-3	0.067	8.21E-3	0.48
beta-Alanine metabolism	21	6	2.21E-3	0.077	8.21E-3	0.46
Alanine, aspartate and glutamate metabolism	28	7	2.25E-3	0.077	8.21E-3	0.62
Arginine and proline metabolism	38	7	2.32E-3	0.077	8.21E-3	0.5
Pantothenate and CoA biosynthesis	19	4	2.55E-3	0.082	8.21E-3	0.02
Sphingolipid metabolism	21	2	2.94E-3	0.091	8.21E-3	0.27
Arachidonic acid metabolism	36	1	3.05E-3	0.091	8.21E-3	0.0
Linoleic acid metabolism	5	1	3.05E-3	0.092	8.21E-3	0.0
alpha-Linolenic acid metabolism	13	1	3.05E-3	0.092	8.21E-3	0.0
Lysine degradation	25	2	3.38E-3	0.092	8.53E-3	0.14
Phenylalanine, tyrosine and tryptophan biosynthesis	4	2	5.34E-3	0.139	0.013	1.0
Selenocompound metabolism	20	1	6.22E-3	0.155	0.014	0.0
Butanoate metabolism	15	3	6.61E-3	0.159	0.014	0.03
Cysteine and methionine metabolism	33	6	8.61E-3	0.198	0.018	0.4

Glyoxylate and dicarboxylate metabolism	32	4	0.011	0.249	0.022	0.15
Taurine and hypotaurine metabolism	8	3	0.012	0.251	0.022	0.43
Biotin metabolism	10	1	0.015	0.300	0.024	0.0
D-Glutamine and D-glutamate metabolism	6	2	0.015	0.300	0.024	0.5
Nitrogen metabolism	6	2	0.015	0.300	0.024	0.0
Porphyrin and chlorophyll metabolism	30	2	0.015	0.300	0.024	0.0
Purine metabolism	66	1	0.022	0.347	0.033	0.0
Tryptophan metabolism	41	4	0.028	0.416	0.041	0.34
Glutathione metabolism	28	6	0.032	0.451	0.046	0.13
Propanoate metabolism	23	2	0.034	0.451	0.047	0.0
Citrate cycle (TCA cycle)	20	1	0.039	0.463	0.052	0.032
Glycolysis / Gluconeogenesis	26	1	0.042	0.463	0.053	0.0
Pyruvate metabolism	22	1	0.042	0.463	0.053	0.0
Tyrosine metabolism	42	1	0.060	0.540	0.071	0.13

The table shows the detailed results from the pathway analysis. Since there are tested many pathways at the same time, the statistical *P* values from enrichment analysis are further adjusted for multiple testing. The Total is the total number of compounds in the pathway; the Hits is the actually matched number from the uploaded data; the Raw *P* is the original p value calculated from the enrichment analysis; the Holm *P* is the p value adjusted by Holm-Bonferroni method; the FDR *P* is the *P* value adjusted using False Discovery Rate; the Impact is the pathway impact value calculated from pathway topology analysis.

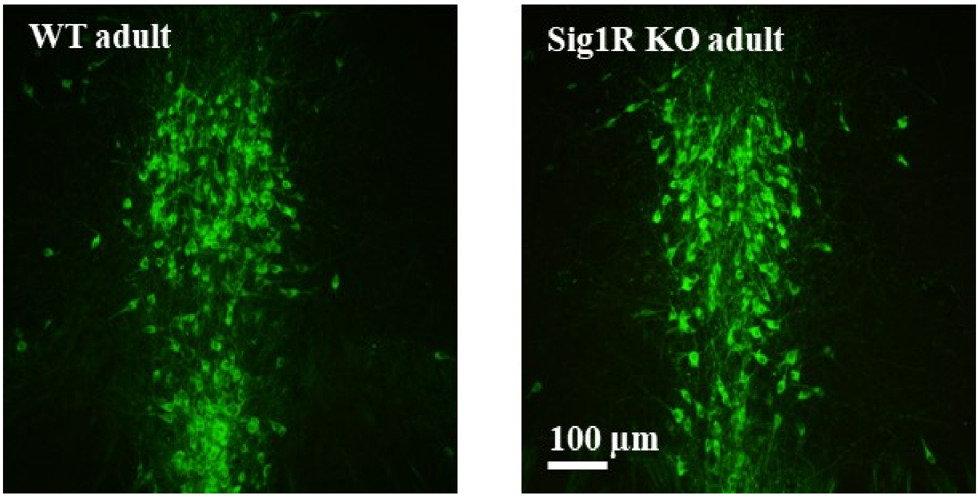

Supplementary Figure S1. Representative images of immunofluorescence staining of TPH2 in adult WT and Sig1R KO animals.
